# Validity of a new sport-specific endurance test in artistic gymnastics

**DOI:** 10.3389/fspor.2023.1159807

**Published:** 2023-04-17

**Authors:** Bessem Mkaouer, Samiha Amara, Raja Bouguezzi, Abderraouf Ben Abderrahmen, Helmi Chaabene

**Affiliations:** ^1^ Department of Individual Sports, High Institute of Sport and Physical Education of Ksar Said, Manouba University, Tunis, Tunisia; ^2^ Department of Physical Education and Sport Sciences, College of Education, Sultan Qaboos University, Muscat, Sultanate of Oman; ^3^ Research Unit (UR17JS01) Sport Performance, Health & Society, Higher Institute of Sport and Physical Education of Ksar Saïd, University of Manouba, Manouba, Tunisia; ^4^ Division of Training and Movement Sciences, Research Focus Cognition Sciences, University of Potsdam, Potsdam, Germany

**Keywords:** artistic gymnastics, field test, aerobic endurance, validity, reliability, assessment, physical fitness, elite athletes

## Abstract

**Introduction:**

General and particularly sport-specific testing is an integral aspect of performance optimization in artistic gymnastics. In artistic gymnastics, however, only non-specific field tests have been used to assess endurance performance (e.g., Multistage Shuttle Run Test; Cooper's Test).

**Methods:**

This study aimed to examine the validity of a new sport-specific endurance test in artistic gymnastics. Fourteen elite-level gymnasts (i.e., eight males and six females) participated in this study. The newly developed artistic gymnastics-specific endurance test (AGSET) was conducted on two different occasions seven days apart to determine its reliability. To assess the concurrent validity of AGSET, participants performed the multistage shuttle run test (MSRT). Maximum oxygen uptake (VO_2max_) and respiratory exchange ratio (RER) were directly assessed using a portable gas analyzer system during both protocols. Additionally, the total time maintained (TTM) during the AGSET, maximum heart rate (HR_max_), maximal aerobic speed (MAS), and blood lactate concentration (BLa) during the two protocols were collected.

**Results:**

The main findings indicated that all variables derived from the AGSET (i.e., VO_2max_, MAS, HR_max_, BLa, and RER) displayed very good relative (all intraclass correlation coefficients [ICC] > 0.90) and absolute (all typical errors of measurement [TEM] < 5%) reliability. Further, results showed that the ability of the AGSET to detect small changes in VO_2max_, MAS, BLa, and RER was good (smallest worthwhile change [SWC_0.2_] > TEM), except HRmax (SWC_0.2_ < TEM). Additionally, results showed a nearly perfect association between the VO_2max_ values derived from the AGSET and MSRT (*r* = 0.985; coefficient of determination [R²] = 97%) with no statistically significant differences (*p*>0.05). The mean (bias) ± 95% limits of agreement between the two protocols were 0.28 ± 0.55 mlminkg-1.

**Discussion:**

AGSET seems to present very good reliability and concurrent validity for assessing endurance performance in elite artistic gymnastics. In addition, the newly developed protocol presents a good ability to detect small changes in performance.

## 1. Introduction

Artistic gymnastics is a demanding Olympic sport that requires high levels of muscle strength, muscle power, strength endurance, flexibility, balance, and aerobic/anaerobic metabolism (1). A typical competition in men's artistic gymnastics requires the athlete to perform six different exercise routines. These are the floor exercise, pommel horse, still rings, vault, parallel bars, and high bar. Women's artistic gymnastics competition consists of four different exercise routines, namely the vault, uneven bars, balance beam, and floor exercise.

It is well-known that the contribution of the various metabolic systems differs from one apparatus to the other (2). In fact, there is evidence that blood lactate concentration (BLa) and heart rate (HR) kinetics depend on the apparatus with the floor and uneven bar exercises inducing the highest values during simulated women's artistic gymnastics competition (3). The same authors also reported that values of HR and BLa were still noticeably higher after 10 min of recovery compared to the resting levels (3). Additionally, Jemni et al. (4) synthesized the literature related to men's artistic gymnastics and concluded that the floor routine is associated with the highest energy cost followed by the pommel horse, rings, high bar, parallel bars, and vault. Furthermore, earlier studies (5, 6) quantified the excess post-exercise oxygen consumption during the first 30 s following female gymnastic routines. Similar to males, results indicated that the floor routine has the highest energy cost followed by the uneven bars, the balance beam, and the vault. Recently, Kaufmann et al. (7) quantified the energetic demands of a simulated floor routine competition in male and female sub-elite artistic gymnasts. The authors demonstrated a predominant contribution of the aerobic system (58.9%) followed by the anaerobic alactic (24.2%) and the anaerobic lactic systems (16.9%). In the same context, Marina and Rodríguez (8) revealed that the average relative peak intensities reached during the various women artistic gymnastics events ranges between 65% and 85% of the individual VO_2max_ and HR_max_ recorded in laboratory conditions. Recently, Goulart et al. (9) examined the energy expenditure and effort intensity during women artistic gymnastics training sessions. They reported that female artistic gymnasts spent higher time at 60%–70% and 70%–80% than at 80%–90% HR_max_. Also, authors revealed that training sessions afford high energy expenditure (768.3 ± 168.5 kcal and 6.1 ± 0.6 metabolic equivalent of task) and concluded that women artistic gymnasts performed long-duration, moderate-intensity training sessions that require high energetic demands.

In this regard, there is clear evidence that the demands on the cardiorespiratory and metabolic systems, either in training and particularly in competition settings, are greater than previously thought. As such, the assessment and development of endurance capacity in artistic gymnastics are crucial for a successful performance. Of note, the development of new valid sport-specific fitness tests that mimic the form of exercise of the sport under scrutiny is warranted (10). Over the last few years, some attempts have been made to create and validate sport-specific tests for artistic gymnastics. Salse-Batán et al. (10) conducted a literature review on the validity and reliability of physical field tests in gymnastics (i.e., artistic, rhythmic, and aerobic gymnastics). The same authors showed that there are only two sport-specific field tests available that assess the aerobic/anaerobic endurance of gymnasts. The first was developed by Alves et al. (11) to assess specific anaerobic endurance performance in elite aerobic gymnasts and the results indicated good reliability (ICC = 0.97) and sensitivity of the protocol. However, this test has some limitations. The test includes only female gymnasts and whenever a gymnast does not complete the skill elements correctly, the test should be canceled. This may prevent athletes from reaching their limits of performance, making the whole protocol rather submaximal. The second was proposed by Gateva (12) to assess the specific endurance of rhythmic gymnasts. The author compared the physiological strain between the specific endurance test and a rhythmic gymnastics competitive routine and revealed similar HR and BLa responses. However, the same author failed to address crucial features of the test, such as validity and reliability, preventing this protocol from being further used by practitioners.

In artistic gymnastics, however, only non-specific field tests have been used to assess endurance performance (e.g., Multistage Shuttle Run Test [MSRT]; Cooper's Test) (10). To the best of the authors' knowledge, there is no sport-specific endurance field test for artistic gymnastics, pointing to a void in the literature. The two above-mentioned sport-specific protocols related to aerobic (11) and rhythmic gymnastics (12) cannot directly be applied in artistic gymnastics. This is because aerobic and rhythmic gymnastics afford different physical and especially physiological demands compared to artistic gymnastics (5). In fact, rhythmic gymnastics demands more joint mobility while artistic gymnastics requires more strength, postural control, and endurance (13). Additionally, artistic gymnastics participation promotes larger hypertrophy of the muscles than rhythmic and aerobic gymnastics (14). In addition, artistic gymnastics requires a higher level of muscle strength and postural control than aerobic gymnastics (15). Therefore, this study aimed to develop and validate a new sport-specific field test to assess specific endurance in men's and women's artistic gymnasts. We specifically aimed to explore the reliability, concurrent validity, as well as sensitivity of the new protocol in elite artistic gymnasts of both sexes.

## 2. Material and methods

### 2.1. Participants

The sample size was estimated *a priori* based on the intraclass correlation coefficient (ICC) using an online calculator tool [https://wnarifin.github.io/ssc/ssicc.html (16, 17)]. We set the minimum acceptable ICC at 0.60, the suspected true ICC at 0.90, the type I error at 0.05, and the statistical power at 80%. The analysis indicated that a minimum of fourteen participants are sufficient. Therefore, fourteen gymnasts, eight males (age 21.56 ± 1.33 years; body height 1.71 ± 0.06 m; body mass 66.11 ± 6.70 kg) and six females (age 20.83 ± 0.75 years; body height 1.68 ± 0.04 m; body mass 58.40 ± 6.95 kg), voluntarily participated in this study. The inclusion criteria included a) regularly competing at the national and/or international level, b) average training volume of 25 ± 2 h per week, and c) being healthy, with no muscular, neurological, or tendon injuries in the last six months. After being informed in advance of the procedures, methods, benefits, and possible risks of the study, each participant had to review and sign a consent form to participate in the study. The experimental protocol was performed per the declaration of Helsinki for human experimentation and was approved by the local ethical committee of the national observatory of sport (ONS/UR/18JS01), Tunis, Tunisia.

### 2.2. Experimental design and procedures

This study is made up of three random assessments (i.e., randomized counterbalanced, Latin Square (18)). Every assessment took place on a separate day with a minimum of 72 h between the artistic gymnastics-specific endurance test (AGSET) and MSRT. Seven days separated the performance of the sport-specific test and retest. Before the first assessment (i.e., 72 h), a familiarization session with AGSET was conducted. All assessments were carried out in the gymnasium at the same time of the day (between 10:00^PM^ and 12:00^PM^). In each of the assessments, the MSRT or the AGSET was conducted. Of note, the AGSET was carried out twice to assess its test-retest reliability. In both protocols, a breath-by-breath portable gas analyzer system was used (Cosmed K4b^2^, Rome, Italy) to quantify the V˙O_2_ and V˙CO_2_ in expired air and the respiratory exchange ratio (RER). The heart rate (HR) was recorded using a Polar Team Pro® heart rate monitor (Polar Electro, Kample, Finlande). A sample of capillary blood from the earlobe was obtained three minutes after the end of MSRT and AGSET for BLa concentration analysis. This was performed using a portable blood lactate analyzer (Lactate Pro® Kit, ARKRAY Factory, Inc. USA).

#### 2.2.1. Multistage shuttle run test

This test involved continuous running back and forth between two lines 20 meters apart at the same time that a sound signal is emitted from a pre-recorded tape. The frequency of the sound signals increases in such a way that the running speed is increased by 0.5 km·h^−1^ each minute from a starting speed of 8.5 km·h^−1^. The test is terminated when the gymnast is no longer able to follow the set pace and failed to reach the 20 m mark twice (19). The last announced stage number or the equivalent maximal aerobic speed (MAS) is then used as the VO_2max_ index. Additionally, the V˙O_2max_, V˙O_2_, and V˙CO_2_ were directly assessed with a breath-by-breath portable gas analyzer system (Cosmed K4b^2^, Rome, Italy).

#### 2.2.2. Artistic gymnastics-specific endurance test

**Figure 1:**
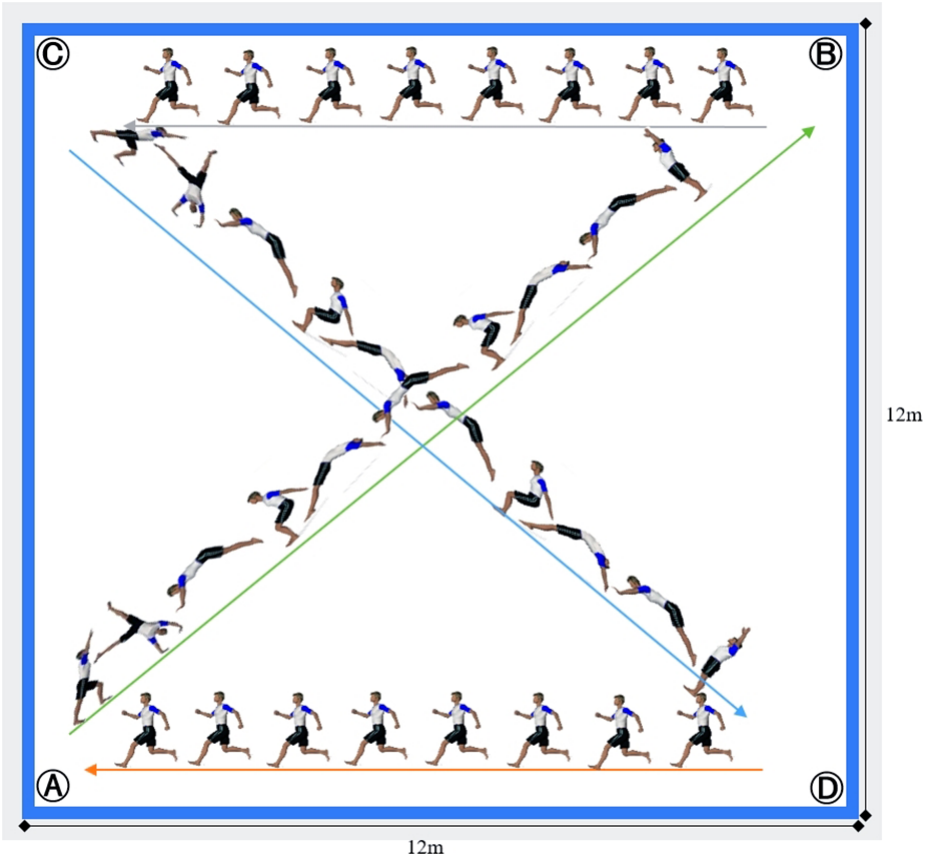
Graphical illustration of the artistic gymnastic-specific endurance test. The test begins with the gymnast standing on the “corner (**A**)”. After the start command (beep), the gymnast performs, in the diagonal, an acrobatic series consisting of a round-off followed by two flic-flac to reach the “corner (**B**)”. At the beep signal, the gymnast runs 12 m on the side of the floor toward the “corner (**C**)”. Following this, and at the beep signal, the gymnast must then perform on the second diagonal a round-off followed by two flic-flac to reach the “corner (**D**)”. After the beep signal, the gymnast then runs 12 m to reach the “corner A”, concluding the first stage. Afterward, the gymnast starts the second stage by repeating the same routine as described above until he/she won't be able to keep up with the pace and/or reaches the corners in time.

The AGSET was inspired by the “Big marathon test” from the FIG women's artistic gymnastics battery (20) which was validated by Nassib et al. (21). The protocol was performed on a regular artistic gymnastics floor (i.e., 14 × 14 m). During each stage of the protocol, the gymnast performs acrobatic elements on the diagonal (i.e., round-off followed by two flic-flac) and runs on the side across 12 m (Figure 1). One complete stage is composed of two diagonals and two sides. The time allowed for each diagonal/acrobatic element is fixed at seven seconds, while the running speed on the sides increased progressively from one stage to the next. The start running speed is 8.5 km·h^−1^ and is increased by 0.5 km·h^−1^ per stage (Table 1). In case the floor corner is reached before the beep sounds, the gymnast must wait until the beep sounds before continuing. However, if the floor corner is not reached before the beep sounds, the gymnast is given a warning and must continue to the floor corner, then turn and try to catch up with the pace imposed by the beeps. A warning was given the first time the gymnast fails to reach the floor corner. The second warning results in an elimination and termination of the protocol. VO_2max_, RER, MAS, HR_max_, BLa, and the total time maintained (TTM) were collected during and following the AGSET for further analysis. The soundtrack (i.e., beeps) was made with Music Maker software version 28.02.43 (MAGIX Software GmbH).

**Table 1:** Details of the artistic gymnastics-specific endurance test.

Speed (km·h^−1^)	Stage	Floor	Time	Total time
8.5	1	Diagonal	7.000	00:00:07:00
		Side	5.082	00:00:12:08
		Diagonal	7.000	00:00:19:08
		Side	5.082	00:00:24:16
9	2	Diagonal	7.000	00:00:31:16
		Side	4.800	00:00:35:96
		Diagonal	7.000	00:00:42:96
		Side	4.800	00:00:47:76
9.5	3	Diagonal	7.000	00:00:54:76
		Side	4.547	00:00:59:31
		Diagonal	7.000	00:01:06:31
		Side	4.547	00:01:10:86
10	4	Diagonal	7.000	00:01:17:86
		Side	4.320	00:01:22:18
		Diagonal	7.000	00:01:29:18
		Side	4.320	00:01:33:50
10.5	5	Diagonal	7.000	00:01:40:50
		Side	4.114	00:01:44:61
		Diagonal	7.000	00:01:51:61
		Side	4.114	00:01:55:73
11	6	Diagonal	7.000	00:02:02:73
		Side	3.927	00:02:06:65
		Diagonal	7.000	00:02:13:65
		Side	3.927	00:02:17:58
11.5	7	Diagonal	7.000	00:02:24:58
		Side	3.757	00:02:28:34
		Diagonal	7.000	00:02:35:34
		Side	3.757	00:02:39:10
12	8	Diagonal	7.000	00:02:46:10
		Side	3.600	00:02:49:70
		Diagonal	7.000	00:02:56:70
		Side	3.600	00:03:00:30
12.5	9	Diagonal	7.000	00:03:07:30
		Side	3.456	00:03:10:75
		Diagonal	7.000	00:03:17:75
		Side	3.456	00:03:21:21
13	10	Diagonal	7.000	00:03:28:21
		Side	3.323	00:03:31:53
		Diagonal	7.000	00:03:38:53
		Side	3.323	00:03:41:85
13.5	11	Diagonal	7.000	00:03:48:85
		Side	3.200	00:03:52:05
		Diagonal	7.000	00:03:59:05
		Side	3.200	00:04:02:25
14	12	Diagonal	7.000	00:04:09:25
		Side	3.086	00:04:12:34
		Diagonal	7.000	00:04:19:34
		Side	3.086	00:04:22:42
14.5	13	Diagonal	7.000	00:04:29:42
		Side	2.979	00:04:32:40
		Diagonal	7.000	00:04:39:40
		Side	2.979	00:04:42:38
15	14	Diagonal	7.000	00:04:49:38
		Side	2.880	00:04:52:26
		Diagonal	7.000	00:04:59:26
		Side	2.880	00:05:02:14

### 2.3. Statistical analyses

Data are reported as means ± standard deviations (SD) and 95% confidence intervals (95% CI). Effect size (*d*) was calculated using GPower software [Bonn FRG, Bonn University, Department of Psychology (22)]. The following scale was used to interpret *d*: < 0.2, (trivial); 0.2–0.6 (small); 0.6–1.2 (moderate); 1.2–2.0 (large); and > 2.0 (very large) (23). The normality of distribution, estimated by the Shapiro-Wilk test, was acceptable for all variables. Therefore, paired sample *t*-test was applied to compare the output of the two protocols (i.e., MSRT and AGSET). The AGSET first test was used for comparison with MSRT. The association between variables derived from both tests was assessed using Pearson correlation coefficients (*r*). The magnitude of the correlation was interpreted as follows: trivial *r *< 0.1, small 0.1 < *r* < 0.3, moderate 0.3 < *r* < 0.5, large 0.5 < *r *< 0.7, very large 0.7 < *r* < 0.9, nearly perfect *r *> 0.9 and perfect *r* = 1 (Hopkins (24).To determine the amount of shared variance, the coefficient of determination (R^2^) was calculated. Additionally, linear regression analysis was conducted to predict the VO_2max_ during the MSRT based on the TTM during the AGSET.

To assess the level of agreement between MSRT and AGSET, the Bland and Altman method was applied (25). Additionally, the relative and absolute reliability of AGSET were examined using the ICC and the typical error of measurement (TEM) expressed as coefficient of variation (CV), respectively. The sensitivity of the AGSET was assessed by comparing the smallest worthwhile change (SWC_0.2_) with the TEM. The SWC_0.2_ was calculated by multiplying the between-subject SD by 0.2 (26). The ability of the test to detect small changes in performance was rated as “good”, “OK”, or “marginal” when the TEM was below, similar, or higher than the SWC_0.2_, respectively (27). The minimal detectable change (MDC_95%_) of the AGSET, which represents 95% CI of the difference in score between paired observations that can be considered real and exceeds measurement errors, was determined as MDC_95%_ = TEM · 1.96 · √2 (28). The significance level was set at *p *≤ 0.05. All statistical analyses were performed using the software package SPSS 20.0 (SPSS, Inc, Chicago, IL, USA) and MedCalc version 9.2.1.0 (Ostend, Belgium).

## 3. Results

The main findings of this study showed no significant differences (*p *> 0.05) in VO_2max_ and MAS between the MSRT and the AGSET (Table 2). Likewise, we observed no significant differences in maximal heart rate (HR_max_), BLa, and RER between the two protocols (*p *> 0.05).

**Table 2:** Comparison between the outcomes of multistage shuttle run test and artistic gymnastics-specific endurance test.

MSRT vs. AGSET		MAG Mean ± SD	WAG Mean ± SD	Total Mean ± SD	*T*-test (*p*)	*d*
HR_max_ (bat·min^−1^)	MSRT	210.21 ± 3.06	198.54 ± 3.26*	204.38 ± 3.96	0.051	0.910
	AGSET	208.32 ± 3.12	196.42 ± 3.52*	200.37 ± 3.97		
Bla (mmol·L^−1^)	MSRT	14.67 ± 0.69	13.22 ± 0.48*	13.95 ± 0.75	0.691	0.099
	AGSET	14.73 ± 0.53	13.02 ± 0.41*	13.87 ± 0.59		
RER (V_CO2_/V_O2_)	MSRT	1.32 ± 0.07	1.27 ± 0.05	1.29 ± 0.08	0.461	0.450
	AGSET	1.35 ± 0.01	1.35 ± 0.01	1.35 ± 0.02		
VO_2max_ (ml·min·kg^−1^)	MSRT	57.13 ± 1.10	49.10 ± 1.19*	53.27 ± 1.28	0.726	0.157
	AGSET	57.20 ± 1.02	49.10 ± 1.23*	53.06 ± 1.09		
MAS (km·h^−1^)	MSRT	13.84 ± 0.40	12.28 ± 0.46*	13.06 ± 0.49	0.945	0.126
	AGSET	13.90 ± 0.45	12.34 ± 0.53*	13.12 ± 0.64		

(MAG) Men's artistic gymnastics; (WAG) Women's artistic gymnastics; (MSRT) multistage shuttle run test; (AGSET) artistic gymnastics specific endurance test; (MAS) maximal aerobic speed; (BLa) blood lactate; (HR) heart rate; (RER) respiratory exchange ratio.

* Significantly different from MAG.

The results of the paired sample t-test indicated no significant test-retest difference for the AGSET (*p *> 0.05), indicative of an absence of systematic bias. The reliability analysis showed that all variables derived from the AGSET (i.e., VO2_max_, MAS, BLa, and TTM) displayed very good relative (all ICC > 0.90 except HR_max_ [ICC = 0.608] and RER [ICC = 0.824]) and absolute (all TEM < 5%) reliability (Table 3). The ICC values were consistent across sexes and ranged between 0.604 and 0.993 (i.e., VO_2max_: 0.993 and 0.964; MAS: 0.976 and 0.956; HR_max_: 0.708 and 0.654; BLa: 0.930 and 0. 824; RER: 0.646 and 0.604, for males and females respectively). Additionally, results showed that the ability of the AGSET to detect small changes in VO_2max_, MAS, BLa, RER, and TTM was good (SWC_0.2 _> TEM), except HR_max_ (SWC_0.2 _< TEM). Furthermore, the MDC_95%_ relative to VO_2max_ and MAS from the AGSET was 0.11 ml.min.kg^−1^ and 0.15 km·h^−1^, respectively.

**Table 3:** Reliability outcomes of the artistic gymnastics-specific endurance test.

Artistic gymnastics-specific endurance test	Test Mean ± SD	Retest Mean ± SD	T-test (*p*)	TEM	TEM _(%)_	MDC _(95%)_	SWC _(0.2)_	ICC _(95% CI)_	*d*
HR_max_ (bat·min^−1^)	200.37 ± 3.97	196.50 ± 1.52	0.051	1.99	1.004	4.518	0.704	0.608 (0.324–0.922)	0.978
Bla (mmol·L^−1^)	13.87 ± 0.59	13.57 ± 0.63	0.124	0.023	0.171	0.065	0.120	0.930 (0.649–0.986)	0.437
RER (VCO_2_/VO_2_)	1.35 ± 0.02	1.34 ± 0.05	0.825	0.019	1.437	0.054	0.080	0.824 (0.120–0.965)	0.196
VO_2max_ (ml·min·kg^−1^)	53.06 ± 1.09	52.95 ± 1.31	0.975	0.077	0.420	0.114	0.234	0.983 (0.914–0.997)	0.085
MAS (km·h^−1^)	13.12 ± 0.64	13.03 ± 0.62	0.711	0.054	0.417	0.150	0.124	0.956 (0.782–0.991)	0.301
TTM (min)	5.58 ± 0.71	5.82 ± 0.72	0.056	0.061	1.065	0.168	0.142	0.957 (0.787–0.991)	0.332

(MSRT) multistage shuttle run test; (MAS) maximal aerobic speed; (BLa) blood lactate; (HR) heart rate; (RER) respiratory exchange ratio; (TTM) total time maintained.

**Figure 2:**
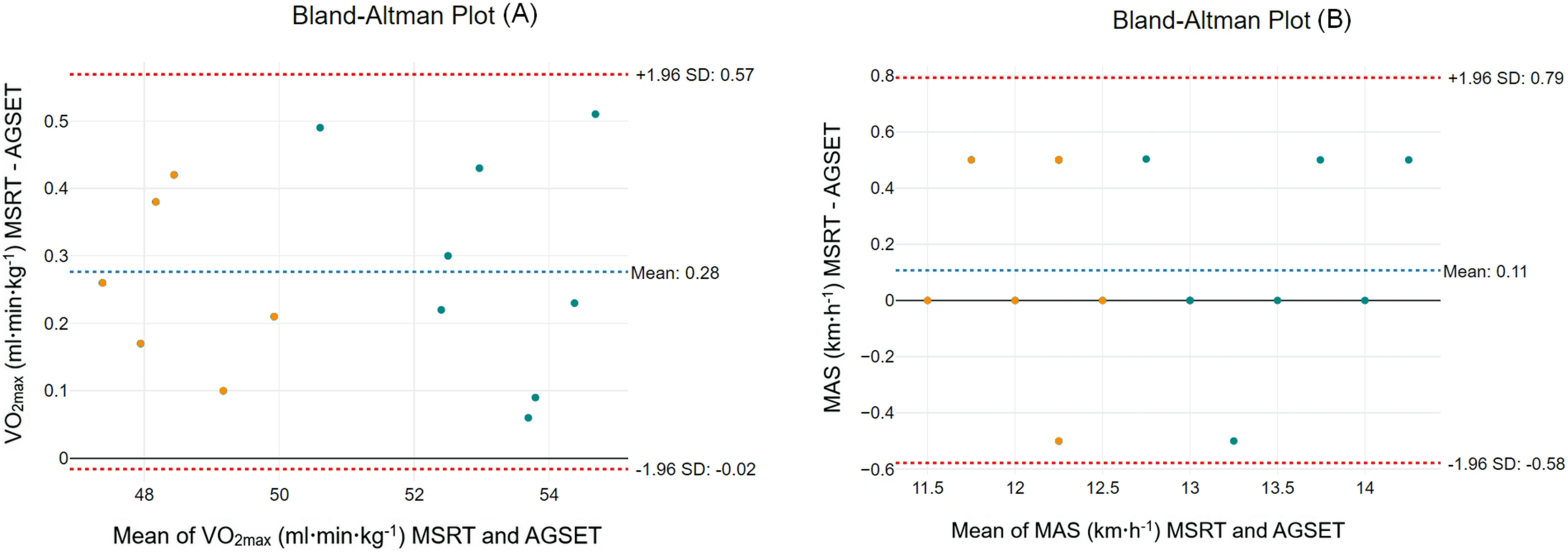
Bland and altman plots for maximal oxygen uptake (**A**) and maximal aerobic speed (**B**) derived from multistage shuttle run test and artistic gymnastics-specific endurance test.

Regarding the correlation analysis, results showed a nearly perfect association between the VO_2max,_ during the AGSET and MSRT (*r *= 0.985 [*R*^² ^= 97%]). Likewise, a nearly perfect association between the MAS registered during the AGSET and MSRT was observed (*r *= 0.984 [*R*² = 0.96]). The mean (bias) ± 95% limits of agreement between the two protocols were 0.28 ± 0.55 ml·min·kg^−1^ for VO_2max_ and 0.11 ± 0.21 km·h^−1^ for MAS (Table 4, Figure 2). Also, large to very large associations between HR_max_ (*r* = 0.830), BLa (*r* = 0.736), RER (*r* = 0.745), and TTM (*r* = 0.889) were noted during AGSET and MSRT. Further, we reported significant, very large correlations between the MAS and VO_2max_ (*r* = 0.984), the BLa and RER (*r* = 0.711), and the TTM and HR_max_ (*r *= 0.886), RER (*r *= 0.923), and MAS (*r *= 0.905) during the AGSET. “Moreover, a significant very large association between AGSET's TTM and VO_2max_ derived from the MSRT was observed” instead Likewise, TTM during the AGSET displayed a significant very large correlation with MAS (*r* = 0.822) from the MSRT.

**Table 4:** Results of the correlation and Bland and Altman analysis between maximal oxygen uptake and maximal aerobic speed derived from the multistage shuttle run test and the artistic gymnastic-specific endurance test.

MSRT vs. AGSET	MSRT Mean ± SD	AGSET Mean ± SD	*r*_(95% CI)_	*R*²	Bias ± 95% LOA
VO_2max_ (ml·min·kg^−1^)	53.27 ± 1.28	53.06 ± 1.09	0.985 (0.920–0.997)	97%	0.28 ± 0.55 ml·min·kg^−1^
MAS (km·h^−1^)	13.06 ± 0.49	13.12 ± 0.64	0.984 (0.879–0.996)	96%	0.11 ± 0.21 km·h^−1^

(MSRT) multistage shuttle run test; (AGSET) artistic gymnastics-specific endurance test; (MAS) maximal aerobic speed; (LOA) limits of agreement.

Linear regression was used to predict the VO_2max_ during MSRT from the TTM during AGSET (equation 1) and VO_2max_ during AGSET from MAS (equation 2). The equation was as follows:

(1) $$MSRT\;VO_{2max}=46.344\;+\;1.20\cdot AGSET\;TTM\;(r=0.810;\;R^{2}=0.604;ESE=0.797)$$

(2) $$AGSET\;VO_{2max}=31.884\;+\;1.629\cdot AGSET\;MAS\;(r=0.794;\;R^{2}=0.601;ESE=0.747)$$

*Where MSRT is the multistage shuttle run test, AGSET is the artistic gymnastics-specific endurance test, TTM is the total time maintained, r is the correlation coefficient, R² is the coefficient of determination, and ESE is the estimated standard error*.

## 4. Discussion

This study aimed to develop and validate a sport-specific field test to evaluate the endurance capacity of artistic gymnasts. The AGSET was created based on the technical and physiological demands of artistic gymnastics routines. The main findings demonstrated that all variables derived from the AGSET (i.e., VO2_max_, MAS, HR_max_, BLa, RER, and TTM) displayed very good relative and absolute reliability. Further, results showed that the ability of the AGSET to detect small changes in VO_2max_, MAS, BLa, RER, and TTM was good, except for HR_max_. Furthermore, results showed a nearly perfect association between the VO_2max_ values derived from the AGSET and MSRT, indicative of a very good concurrent validity of the new protocol to assess endurance performance in elite artistic gymnasts.

Aerobic capacity is crucial for successful performance in artistic gymnastics. In this sense, a recently published study by Kaufmann et al. (7) demonstrated that aerobic metabolism predominates the energy produced (58.6%) during the floor routine followed by the anaerobic alactic (24.2%) and the anaerobic lactic (16.9%) systems in male and female sub-elite gymnasts. In fact, artistic gymnastics is a high-intensity intermittent sport, in which the acrobatic routine during the floor exercise can last up to 90 s (5). It is therefore conceivable that performance is largely dependent on the energy transferred through aerobic metabolism, but also anaerobic systems (7). Artistic gymnastics develops more strength, power, speed, and endurance than other gymnastic disciplines (13). Women's artistic gymnastics has less relative explosive power and strength than men's artistic gymnastics, specifically in the upper body (29). Also, men's artistic gymnastics reaches 8%–9% higher run-up speeds than women's artistic gymnastics (29). Sex differences in sprint speed, strength, and power are of a biological origin, and they do not affect the energetical demands of artistic gymnastics (6). The results of the present study showed high relative and absolute reliability of the different parameters derived from the AGSET. Specifically, the ICC and TEM values calculated between the test and retest for VO_2max_, and MAS were >0.90 and < 5%, respectively. Further, the sensitivity of AGSET was assessed by comparing the SWC_0.2_ with the TEM. Results showed that the ability of the AGSET to detect small changes in VO_2max_, MAS, BLa, RER, and TTM was good (SWC_0.2 _> TEM), except for HR_max_ (SWC_0.2 _< TEM). Moreover, the MDC_95%_ is a useful metric that outlines the relative change necessary that would reflect a “true” (i.e., significant) enhancement in performance beyond the random changes. The MDC_95%_ value for VO_2max_ showed in this study was 0.11 ml·min·kg^−1^ indicating that a change in the VO_2max_ beyond this value could be considered “real” and reflect a true performance improvement in elite-level artistic gymnasts. This study is unique and therefore there are no previous findings to compare with. Of note, there are only two specific endurance tests available. The first was developed by Alves et al. (11) for aerobic gymnastics and the second was developed by Gateva (12) for rhythmic gymnastics (10). Besides the methodological concerns (12), the two protocols cannot be used with artistic gymnasts because of the different physical and physiological features that artistic gymnastics affords compared with aerobic and rhythmic gymnastics (5). Rhythmic gymnastics develops more joint mobility and artistic gymnastics develops more strength, balance, and endurance (13). There is partial use of the lower limbs in rhythmic gymnastics and bilateral improvement in the upper limbs in artistic gymnastic athletes (30). Thus, artistic gymnastics seems to promote more muscle hypertrophy than rhythmic and aerobic gymnastics do (14). In addition, artistic gymnastics develops more strength and balance than aerobic gymnastics (15). Therefore, the physiological adaptations elicited by artistic, rhythmic, and aerobic gymnastics are likely different. With this in mind, the current sport-specific field protocol is the first for artistic gymnastics. As such, future studies including a larger sample size are needed to substantiate the present findings and to establish normative values for artistic gymnastics.

The concurrent validity of AGSET was assessed by establishing its association with the MSRT. The main results demonstrated a nearly perfect correlation with a high level of shared variances between the VO_2max_ and MAS derived from both measurement protocols (*r *= 0.985 [*R*^² ^= 97%] and *r *= 0.984 [*R*² = 0.96], respectively). This indicates that the newly developed protocol can assess key parameters of the aerobic system including the VO_2max_, which is generally accepted as the best single measure of aerobic performance (31). Additionally, the mean (bias) ± 95% limits of agreement between the two protocols were 0.28 ± 0.55 ml·min·kg^−1^ for VO_2max_ and 0.11 ± 0.21 km·h^−1^ for MAS. The small mean differences between the AGSET and MSRT confirm the results of the correlation analysis, that the new sport-specific protocol is valid to assess endurance performance in artistic gymnasts of both sexes. Interestingly, the TTM during the AGSET displayed a significant, very large association with MAS from the MSRT (*r *= 0.822). This means that TTM during the AGSET can be used as a proxy for the endurance performance of artistic gymnasts.

### 4.1. Limitations

This study is not without limitations. First, the recruited small sample size (*n* = 14) is relatively small. However, unlike team sports, the overall athletic population in artistic gymnastics is rather small, so attempting to recruit a larger sample size is very difficult or even unrealistic, particularly at the elite level. Nevertheless, future studies with larger sample sizes are needed to substantiate the current findings. Second, it would have been better to use the treadmill laboratory test, as the gold standard, in addition to the field test. However, this was not affordable at the time. Additionally, the MSRT is a well-accepted valid and reliable field test that has been used in different populations, regardless of age, sex, and level of experience (32, 33). Furthermore, we opted to directly measure the gas exchange during the MSRT using a portable gas analyzer system and this would account for the absence of a treadmill laboratory test.

## 5. Conclusions

Based on the current findings, the AGSET could be considered a valid and reliable tool to assess aerobic endurance in high-level artistic gymnasts. Practitioners who do not afford to measure VO_2max_ directly e.g., using a portable gas analyzer system, can rely on MAS and/or the TTM as informative proxies for endurance performance in high-level artistic gymnasts.

## Data Availability

The original contributions presented in the study are included in the article/Supplementary Material, further inquiries can be directed to the corresponding author/s.
